# Canaries’ Microbiota: The Gut Bacterial Communities along One Female Reproductive Cycle

**DOI:** 10.3390/microorganisms11092289

**Published:** 2023-09-11

**Authors:** Jasmine Hattab, Giuseppe Marruchella, Alessandra Sibra, Pietro Giorgio Tiscar, Gianluca Todisco

**Affiliations:** 1 Department of Veterinary Medicine, University of Teramo, SP18 Piano d’Accio, 64100 Teramo, Italy; jhattab@unite.it (J.H.); gmarruchella@unite.it (G.M.); 2 APHA—Animal & Plant Health Agency, Building 1, Sevington Inland Border Facility, Ashford TN25 6GE, UK; alessandra.sibra@apha.gov.uk; 3 Independent Researcher, 72017 Ostuni, Italy; todvet@yahoo.it

**Keywords:** canaries, *Serinus canaria*, gut bacterial microbiota, bacterial communities, reproduction, reproductive cycle, 16S rRNA gene sequencing

## Abstract

Investigations of bacterial communities are on the rise both in human and veterinary medicine. Their role in health maintenance and pathogenic mechanisms is in the limelight of infectious, metabolic, and cancer research. Among the most considered, gut bacterial communities take the cake. Their part in animals was assessed mainly to improve animal production, public health, and pet management. In this regard, canaries deserve attention, being a popular pet and source of economic income for bird-keepers, for whom breeding represents a pivotal point. Thus, the present work aimed to follow gut bacterial communities’ evolution along on whole reproductive cycle of 12 healthy female canaries. Feces were collected during parental care, molting, and resting phase, and submitted for 16S rRNA sequencing. Data were analyzed and a substantial presence of *Lactobacillus aviarius* along all the phases, and a relevant shift of microbiota during molting and rest due to an abrupt decrease of the Vermiphilaceae family were detected. Although the meaning of such change is not clear, future research may highlight unforeseen scenarios. Moreover, *Lactobacillus aviarius* may be deemed for normal bacteria flora restoration in debilitated birds, perhaps improving their health and productivity.

## 1. Introduction

The gut microbiota is regarded as a full-fledged endocrine organ because of its numerous effects on distant organs and pathways [1]. Commensal bacteria can produce and secrete hormones, and the interaction between hormones and microbes impacts the metabolism, immunity, and behavior of the host. Changes in the microbiota, particularly in the gut microbial communities, have specific effects on the reproductive endocrine system [2]. In this respect, metagenomic techniques development offered a priceless opportunity to unveil microbial ecology. Among the available technologies, 16S rRNA gene sequencing represents an effective and economically affordable solution, yielding the identification of the genus level of most bacteria characterizing an environment [3,4]. Human medicine greatly took advantage of the application of metagenomics, enhancing the comprehension of microbes-host interactions and learning how to modulate microbial communities’ composition for health purposes. In women, microbiota imbalance was linked to several disease conditions, from cancer to reproductive issues such as endometriosis, polycystic ovary syndrome (PCOS), pregnancy complications, and adverse pregnancy outcomes [1]. The correlation between the shifts in the gut bacterial communities and reproduction was investigated in many animal species, finding connections between microorganisms and the endocrine system of their host. Animals have complex and species-specific reproductive interactions that are finely tuned, and gut bacterial microbiota was demonstrated to greatly affect physiology and behavior by impacting neurotransmitters and neuropeptides [5]. As regards birds, gut bacterial microbiota was investigated in a variety of captive and wild avian species, focusing mostly on the interplay between the gut microbiota composition and specific bacteria (especially pathogens), diet, season, and migration of the avian host [6,7]. The relationship between microbiota and reproduction was explored mainly in laying hens and endangered birds, for commercial and conservation respectively confirming the gut bacterial community’s footprint on reproductive performances [8,9]. In a study on finches (*Lonchura striata domestica* and *Taeniopygia guttata*), consistent differences were found between individuals, with more similarity being observed within mating pairs in comparison with out-of-the-couple birds. Furthermore, more significant shifts were observed in males with respect to females. This finding was explained based on testosterone fluctuation in males between the breeding and non-breeding seasons [10].

Canaries (*Serinus canaria*) are Fringillidae songbirds appreciated for their voice, colors, and gentle nature. Kept as a pet and increasingly popular, they are receiving more and more attention, making their breeding profitable [11,12]. Canaries are non-migratory birds, whose reproductive cycle is composed of three phases: winter/nonbreeding, breeding, and molt [13]. Reproductive disorders of canaries include egg-binding, dystocia, ovarian cysts, and bacterial infections. *Klebsiella*, *Escherichia*, *Pantoea*, *Bacillus,* and *Staphylococcus* are reported as the main responsible for bacterial disease conditions [14,15]. Most studies on canaries’ reproduction focused on social behavior, neurodevelopment, and the effects of sex hormone alteration on song development [16,17].

So far, few data are available on the gut microbial communities of canaries (*Serinus canaria domesticus*), and little is still known about the reproductive health of female canaries in relation to the complex microbiota interactions of their lower reproductive tract [18,19]. Exploring the microbial pathways and determining the variables that affect the bacterial communities, in either a positive or negative way, may be a turning point in canaries’ management. Implications for the wellbeing of the female individuals, their reproductive health, and the microbiological condition of their offspring should be considered. For instance, the setup of microbiological markers used as a diagnostic tool to screen the canaries may help prevent or highlight possible sub-clinical conditions. Also, an ad hoc pro- or prebiotic formulation may be designed to achieve the re-establishment of a healthy microbiota, as recently proposed in human medicine [20]. Thus, this study aimed to provide a first description of the gut bacterial communities of healthy female canaries throughout one whole reproductive cycle, evaluating possible shifts in microbial communities between each phase and posing the basis for health instrument development.

## 2. Materials and Methods

### 2.1. Sampling

A total of 12 female *Serinus canaria domesticus* were included in the study. They were all color canaries, aged between 18 and 24 months. The breeding group consisted of 120 canaries housed in battery cages (60 × 32 × 40 cm). The environmental temperature was controlled in winter, always above 15 °C, with 55–70% relative humidity. The canaries were fed with commercial seed mash. Supplements containing vitamins, mineral salts, and cuttlefish bones were given during the mating period, while polyunsaturated fatty acids (PUFA) were added to the diet during the molting period. Antibiotics were administered only when the disease occurred, and the bacteriological origin was identified. In such cases, a bacteriological culture and an antibiogram were performed to select the most appropriate therapy. No antibiotics were given during the trial. A clinical evaluation of birds in the cage was performed by an experienced physician on all the involved subjects before each sampling. Quality of the feathers, nares, beak, eyes, vent, and feet were regarded as criteria for health assessment. The canaries were sampled three times between July and November 2022. The first sample was taken during the parental case phase, the second during the molting period, and the third during the resting phase before the start of a new reproductive cycle. A total of 35 samples were collected, as one of the canaries died before the last sampling. (Table 1).

Prior to each collection, dry heat-sterilized waxed paper was placed on the bottom of the cages. Freshly deposited feces were collected from the waxed paper using a disposable sterile scalpel blade (a new blade was used for each collection) and transferred to cryogenic vials (Thermo-Fisher Scientific, Waltham, MA, USA). The vials were immediately placed in a cryo-container filled with liquid nitrogen to prevent sample alteration.

### 2.2. DNA Extraction

Total genomic DNA was extracted under a laminar flow cabinet. A commercial kit for DNA isolation was used according to the manufacturer’s instructions (Exgene™ Stool DNA mini, Seoul, Republic of Korea) and stored at −20 °C until use. DNA concentration was assessed by Qubit fluorometer (Invitrogen, Carlsbad, CA, USA), and samples were normalized at 10 ng/µL concentration.

### 2.3. 16S rRNA Sequencing

V3–V4 region of the 16S rRNA gene was amplified using the following primers: F, 5′-CCTACGGGNGGCWGCAG-3′, and R, 5′-GACTACHVGGGTATCTAATCC-3′. Primers were modified with forward and reverse overhangs (Forward overhang: 5′-TCGTCGGCAGCGTCAGATGTGTATAAGAGACAG-[locus specific sequence]; Reverse overhang: 5′-GTCTCGTGGGCTCGGAGATGTGTATAAGAGACAG-[locus specific sequence]) necessary for dual index library preparation. For more details see the Illumina MiSeq protocol (16S Metagenomic Sequencing Library protocol n. 15,044,223 Rev. B). Sequencing was performed on Illumina MiSeq (San Diego, CA, USA) using a 2 × 300 flow cell V3 chemistry.

### 2.4. Data Analysis

Bacterial microbiota analysis was performed with QIIME 2 2021.11 [21]. Q2 demux plugin was used to demultiplex raw sequences. Quality filter was applied by means of the q2-demux plugin and denoising was carried out with DADA2 via q2-dada2 [22]. The amplicon sequence variants (ASVs) were then aligned via q2-alignment with mafft [23]. Aligned sequences were used to produce an approximately maximum-likelihood phylogenetic tree with FastTree2 via q2-phylogeny [24]. Alpha-diversity metrics, namely Chao1, Faith’s Phylogenetic Diversity, Evenness, Observed Features, and Simpson and Shannon Indexes were used [25,26,27,28,29]. Beta diversity metrics were estimated to assess differences between groups A, B, and C. In particular, weighted UniFrac [30], unweighted UniFrac [31], Jaccard distance, and Bray-Curtis dissimilarity [32,33], were obtained using q2-diversity. All the Alpha and Beta diversity indexes were computed based on the genus level. Silva v138.1 was used as a reference for taxonomic annotation of ASVs [34,35]. Classification of the reads had 0.96 precision to the genus level, Recall of 0.93, and F-measure of 0.95. Statistical computing and visualization were performed in the R v4.1 environment [36]. Permutational multivariate analysis of variance (PERMANOVA) test was used to evaluate differences in gut bacterial communities between groups based on 1000 permutations [37]. Results were considered statistically relevant when the *p*-value was below 0.05.

## 3. Results

### 3.1. Sequencing Results and GBC Composition

**Figure 1:**
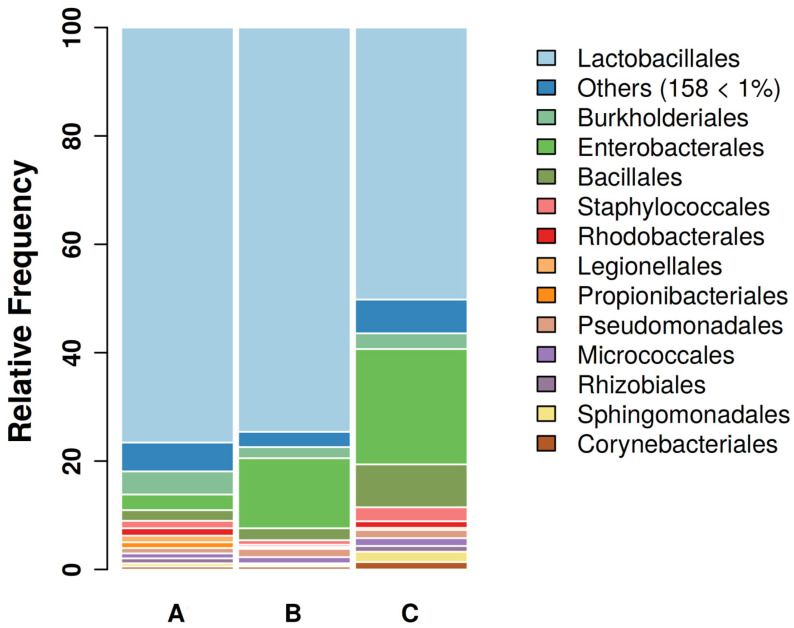
Gut bacterial communities of canaries’ feces at the order level. Bar plots showing the main bacterial composition of the female canaries’ fecal community during parental care (A, 12 samples), molting (B, 11 samples), and resting phase (C, 11 samples) at the order level. Only orders with relative abundance >1 are shown singularly.

A total of 34 samples were included in the final analysis, due to insufficient DNA amount in one sample. Thus, groups A, B, and C consisted of 12, 11, and 11 samples, respectively. From a minimum of 12,126 to a maximum of 104,841 features per sample were observed, with a total frequency of 2,133,870. In general, 4179 sequences were identified, with an average length of 392.08, a minimum length of 273, and a maximum length of 448. Globally, 171 orders were assigned within the total samples. The most abundant orders were Lactobacillales (68.96%), Enterobacterales (11.64%), Bacillales (3.67%), Burkholderiales (3.10%), and Staphylococcales (1.50%), accounting for 88.87% of the total reads (Figure 1).

**Figure 2:**
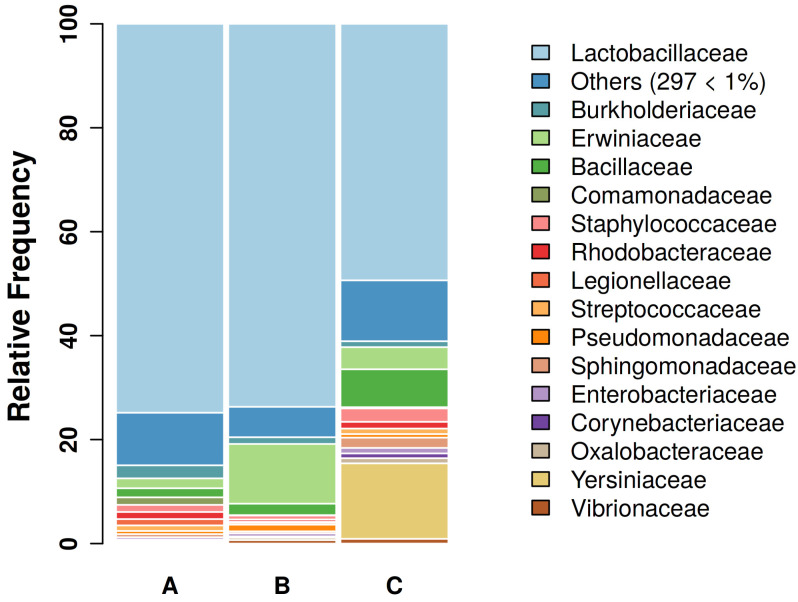
Gut bacterial communities of canaries’ feces at the family level. Bar plots showing the main bacterial composition of the female canaries’ fecal community during parental care (A, 12 samples), molting (B, 11 samples), and resting phase (C, 11 samples) at the family level. Only families with relative abundance >1 are shown singularly.

A total of 333 families were identified. The families with the highest relative abundance were Lactobacillaceae, Erwiniaceae, Yersiniaceae, Bacillaceae, Burkholderiaceae, Staphylococcaceae, Rhodobaceraceae, Pseudomonadaceae, Sphingomonadaceae, and Streptococcaceae (Figure 2).

At the genus level, 787 genera were found, with *Ligilactobacillus*, *Pantoea*, *Serratia*, *Bacillus*, *Staphylococcus*, *Ralstonia*, and *Pseudomonas* being the most observed. In terms of identified species, *Ligilactobacillus aviarius*, formerly *Lactobacillus aviarius* [38], was by far the most represented, its feature being found 1,363,443 times out of a total of 2,133,870 global features (63.89%). Lactobacillales were found in all 34 examined samples, and *L*. *aviarius* in 32 out of 34 samples.

### 3.2. Alpha Diversity

**Figure 3:**
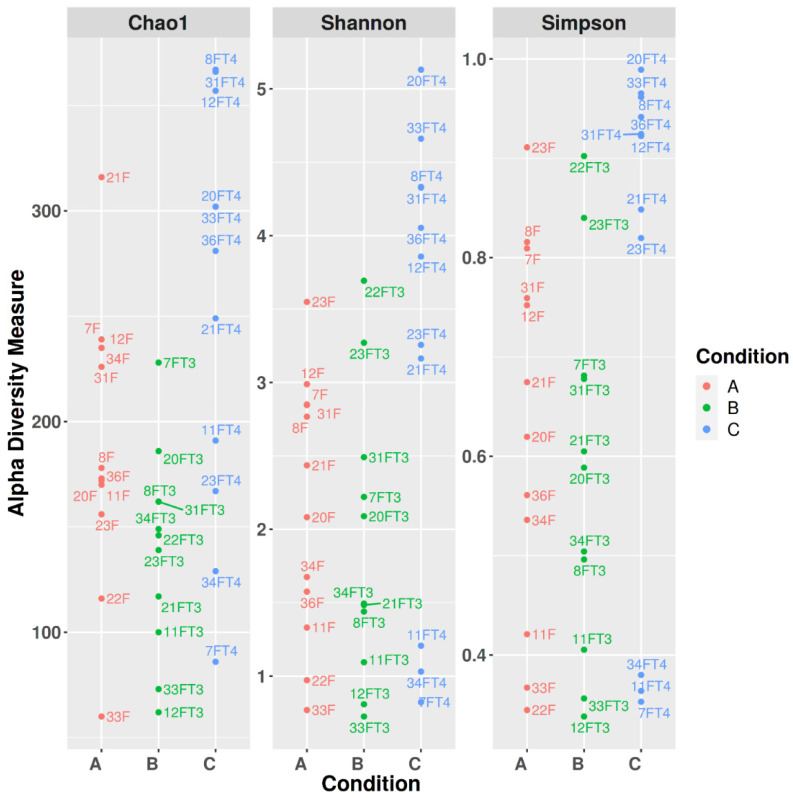
Alpha diversity of gut bacterial communities of female canaries along one reproductive cycle is shown according to Chao1, Shannon, and Simpson’s indexes calculated at the genus level. Observed genera and microbiota diversity are represented in the Chao1 graphic, and combined genera and abundance are shown in the Shannon and Simpson graphics. Data are divided according to reproductive phase, i.e., parental care (A), molting (B), and resting phase (C).

Alpha diversity was assessed by means of Chao1, Shannon, and Simpson’s indexes (Figure 3).

Pielou’s Evenness, Faith phylogenetic diversity, Observed Features, and Shannon indexes were used to assess phylogenetic dissimilarity within and between the groups (Table 2).

The comparison of the obtained values yielded *p*-values respectively of 0.121, 0.013, 0.006, and 0.055 (*p* < 0.05 was considered statistically significant). More in detail, Pielou’s Evenness index comparison between groups A, B, and C suggests that there is a statistically relevant difference in the number and abundance of the taxa between the communities, only when comparing A and C (*p* = 0.048), (A vs. B 0.622, B vs. C 0.122). As concerns Phylogenetic diversity (Faith), the phylogenetic distance between the communities belonging to the groups was significant. In particular, group B clustered separately from A and C, having a lower phylogenetic distance between its community components than the other two groups (A vs. B 0.026, A vs. C 0.218, B vs. C 0.009). When considering observed features (i.e., richness within each group), B richness is lower than the other groups, especially lower than C (*p* = 0.009), (A vs. B 0.022; A vs. C 0.056; B vs. C 0.009). Shannon diversity, which accounts both for diversity and relative abundance of the taxa composing a community, showed a trend in diversity between groups. Pairwise comparison highlighted meaningful differences when comparing A and C, and B and C (A vs. B 0.423; A vs. C 0.042; B vs. C 0.045). Briefly, the group clustering more separately from the others is C, which showed statistically significant differences, especially when compared to B.

### 3.3. Beta Diversity

**Figure 4:**
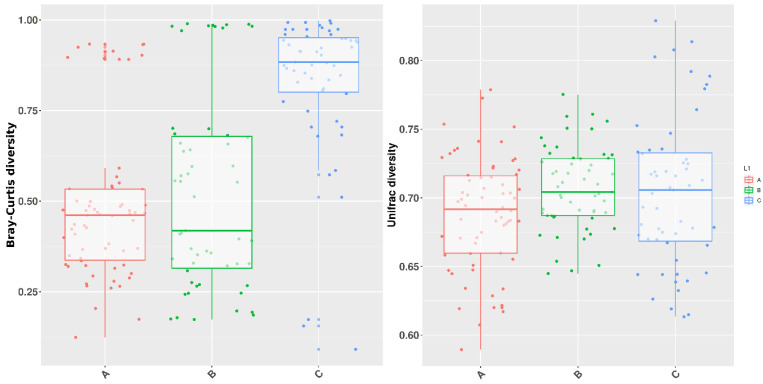
Box and whiskers plots illustrating the Beta diversity based on Bray-Curtis and Unweighted Unifrac diversity calculated at the genus level. Groups are represented in different colors, each point being the graphical representation of the distance comparison among the three groups A, B, and C. The lower whiskers represent the values from the minimum value up to the first quartile, and the upper whiskers the values ranging from the third quartile and the maximum value.

Beta diversity significance was estimated through Bray-Curtis dissimilarity and Unweighted Unifrac by Permanova analysis (Figure 4).

Comparisons according to Bray-Curtis dissimilarity yielded a global *p*-value equal to 0.004 among the three groups, while in single comparisons, the major distance of C with regards to the other groups was more striking (A vs. B 0.863; A vs. C 0.002; B vs. C 0.011), meaning that group C has a more different community composition with respect to A and B. Unweighted Unifrac *p*-value was equal to 0.0009, stating a meaningful difference between the overall composition of the three groups (pairwise results A vs. B 0.001; A vs. C 0.005; B vs. C 0.003), thus accounting both for phylogenetic distance and presence of taxa (Table 3).

The difference between the three groups is attributable to the highest relative abundance of Legionellales and Babeliales at the order level in group A, followed by a mild presence in group B and very few in group C (W 133 and 122, respectively). Vermiphilaceae’s presence in the three groups followed the same pattern as Babeliales at the order level (W 250). In total, 10 out of 13 samples containing Vermiphilaceae belonged to group A, and the remaining 3 were specimens collected from individuals who tested positive for the same family during the parental care phase sampling. More in detail, one canary showed the presence of Vermiphilaceae during parental care and molting phases (1.359% vs. 0.053%), and another during all three phases (6.183% vs. 0.190% vs. Vermiphilaceae < 0.05%). The same was assessed at the genus level (Vermiphilaceae *undetermined genus*, W 603). At the genus level, *Proteus* also turned out to be determined in the statistical difference between groups, its feature being consistently more observed in group C (W 535). Globally, *Proteus* sp. was found in 10 out of 34 samples, 8 of which belonged to group C (resting phase), and 2 to group A (parental care phase). The two samples that proved positive for *Proteus* sp. presence during the parental care phase were positive during the molting phase, and while one showed a reduction in *Proteus* sp. relative frequency (0.050% vs. 0.021%), the other showed an increase (0.102% vs. 2.611%).

## 4. Discussion

The present study provides robust data on the gut bacterial communities of healthy female canaries throughout one reproductive cycle. It was observed a significant shift between three reproductive phases (i.e., parental care, molting, and resting phase).

In all the samples examined, Lactobacillaceae was the most consistent family of the gut bacterial microbiota. Lactobacillaceae are recognized as a relevant component of the fecal and cloacal bacterial microbiota in avian hosts, and they have been found to have a major role in the gut microbiota of many vertebrates. In humans, Lactobacillaceae represent 1–2% of the overall distal gut population, and despite not being as numerous as in other organisms, species and genotypes belonging to the genus *Lactobacillus* were proposed as gut health biomarkers [39,40]. Notably, in this study, a consistent part of the bacterial communities observed was composed of *L*. *aviarius*. The presence and high percentages of *L*. *aviarius* in almost all the examined samples suggest its common presence in canaries’ feces and designate it as the main component of the “core” bacterial microbiota. In general, such bacterial components are regarded as a marker of a healthy community [41]. According to studies carried out in vitro, a relationship was suggested between the *Lactobacillus* genus and an improved intestinal barrier function, both due to the increased mucin secretion and the promotion of goblet cell proliferation [42,43]. *L. aviarius* was first isolated from the intestine of chickens [44]. Since then, it was demonstrated to be among the most abundant gut microbiota components broilers [45], laying hens [46], chickens [47,48], and turkeys [49]. It was also recognized as a part of the core fecal microbiota of the takahe (*Porphyrio hochstetteri*), an endangered New Zealand bird [50]. *L. aviarius*’ role in the gastrointestinal bacterial community of avian species is still unclear and even controversial. In fact, some studies state a relationship between *L. aviarius* abundance and body-weight gain or feed conversion [48,51,52], which may be related to an increased absorption area induced by Lactobacilli [46]. *L. aviarius* abundance was also associated with an enhanced mycotoxins clearance in broilers [53]. Nonetheless, this species may be responsible for an increase in the intestinal mucosa permeability in laying hens [54], and another work found a negative correlation between a high *L. aviarius* relative abundance and growth in turkeys [49]. In a nutshell, the presence of *L. aviarius* and its high relative abundance in many avian species suggests a prominent role in the GI ecology of birds. Nevertheless, much remains to be unveiled about its function and balance in relation both to the host and the rest of the GI bacterial species [47].

*L*. *aviarius* could be carefully considered as a benchmark for healthy gut bacterial communities of canaries though, as mentioned previously, more specific studies for this species are needed. Our findings on gut bacterial communities’ composition are consistent with previous studies carried out on many avian species, including pheasants, parrots, and chickens [40,55]. In canaries, fecal bacterial microbiota was analyzed in two papers. The first was carried out on 6 canaries’ flocks of pooled feces, and the family Lactobacillaceae was observed in all the examined flocks and ranged approximately from 10% to 90% of the overall families. Such variability was attributed to diet variations between flocks [18]. The second was carried out on 44 canaries from the same breed in relation to *Macrorhabdus ornithogaster* infection. The genus *Lactobacillus* was found to be more abundant in infected birds than in uninfected ones (32% vs. 6%), maybe due to an infection-dependent increase in gastric pH, which possibly favored *Lactobacillus* proliferation [19]. In the present study, all the sampled canaries were clinically healthy, and the same feed was administered to all of them throughout the study. Other studies were carried out to investigate the gut microbiota of pet birds, and in most cases, Lactobacillaceae represented the most relatively abundant bacterial component of feces (Table 4).

When analyzing Alpha diversity indexes, higher values were observed for group C (resting phase), especially with respect to B (molt), which showed the lowest Alpha diversity index values. The reduction in gut bacterial communities’ phylogenetic diversity (Faith), observed features, and relative abundance (Table 1) during this phase can be ascribed to the physiological alterations that come with the molt. More in detail, changes in thyroid hormones, gonadal steroid hormones, and prolactin are involved in the molting process. Among hormones, prolactin seems to play a major role, decreasing gradually along with the light hours and eventually triggering the start of the post-breeding molt. Increase in basal metabolism with respect to non-molting periods, protein synthesis, bone and lipids metabolism, and immune system functionality are affected during molt [59,60,61,62]. Feather replacement and changes in tissue metabolism are the main features of molt, which make it energy-consuming for the avian host. In many species, molt is avoided during periods of high energy demand, and for this reason it generally follows the reproductive phase [63]. Molt was linked to alterations in the gut bacterial communities’ composition, and a shift towards potentially pathogenic bacteria is reported both in wild birds and poultry. Such changes depend on a reduction in light hours and on the fasting/caloric restriction laying hens and wild birds face during molt [64,65,66]. In pet passerines, which do not undergo feed reduction, molt starts in response to changes in daylight hours [67]. Thus, the changes in gut bacterial microbiota observed in the present studies were probably related to molt per se.

Beta diversity analysis showed significant differences among groups. The diversity pattern was similar to Alpha diversity, although the gap between groups was even more pronounced, both when considering Bray-Curtis dissimilarity (*p* ≤ 0.01 in comparisons involving group C) and Unweighted Unifrac (*p* ≤ 0.005 in all groups comparisons). Therefore, communities’ composition was more different in group C, and the combination of taxonomic composition and phylogenetic distance were significantly different between all three groups. A more diverse microbiota has been associated with a better health status of the host. In fact, more diverse ecosystems have a certain degree of redundancy which allows compensation of function whenever a species is lost or removed [68]. The findings of the present study could be suggestive of the importance of the resting period for restoring an optimal bacterial microbiota of the host before the start of a new reproductive cycle.

Regarding gut communities’ composition, differences among groups were largely due to a marked decrease in the orders Babeliales and Legionellales during the molt and resting phase. Legionellales were so far found mostly in invertebrates’ gut microbiota, such as clams and ascidians. In the latter, Legionellales are possibly involved in compensative mechanisms during starvation [69,70]. On the other hand, Legionellales are globally distributed in the environment, being found in soil, freshwater, and seawater. Nonetheless, little is known about the species and diversity of the bacteria belonging to this order. Apart from pathogenic members of Legionellales, other species have received little attention, were not sequenced, and went unnoticed in 16S rRNA analysis [71]. Within Babeliales order, the family Vermiphilaceae decrease was responsible for the shift in microbiota composition. Vermiphilaceae family has been mentioned so far in studies investigating the gut microbiota of lizards (*Sceloporus* spp.), giant river prawn *Macrobrachium rosenbergii*, and ascidian (*Halocynthia roretzi*), which was put in relation to age, growth rate and season respectively [70,71,72,73]. Nevertheless, little is known about its role in the gut bacterial microbiota dynamics and its ecology in living host communities. At the genus level, an increase in *Proteus* relative abundance during the resting phase was observed. Globally, *Proteus* sp. was found mostly in group C (8 out of 10 samples positive for *Proteus* sp.). In two canaries, *Proteus* sp. was observed both during the parental care phase and during the resting phase, but no single individual showed its presence during molt. The restoration of a genus during the resting phase with respect to the parental care stage, along with an increase in Alpha and Beta diversity could suggest the re-establishment of the gut bacterial microbiota after molt. *Proteus* spp. are regarded as common commensals of the gastrointestinal tract microbiota, and in avians, the presence of *Proteus* sp. was assessed in the gut microbiota of clinically healthy bird species including passerines and psittacines [74,75,76,77,78].

In general, shifts along the reproductive cycle were observed in many passeriformes. In tree swallows (*Tachycineta bicolor*), microbiota changes between nest building and incubation, and in rufous-collared sparrows (*Zonotrichia capensis*) fluctuations of cloacal microbiota composition were associated with the breeding condition of the host [79,80]. Our findings are consistent with the available literature, although much land remains to be conquered on microbiota composition in avian hosts.

As regards supplement administration, the action of dietary intake of PUFA on the gut microbiota is uncertain. PUFA are regarded as prebiotics by some authors, while other studies stated that dietary intake of fatty acids may change the fatty acid composition of the gut wall and therefore alter the attachment site of bacteria, promoting or inhibiting microbial colonization [81,82]. Lastly, other researchers found no correlation at all between PUFA administration and microbiota shifts [83]. In the present study, canaries belonging to the examined flock were routinely given dietary PUFA supplementation as an aid for feather regrowth during molt. Thus, all the canaries involved in the study received PUFA, and no control group was made to examine the effect of dietary augmentation of fatty acids on gut microbial communities. Nevertheless, it was not the aim of the present investigation, although it would be interesting to assess the possible impact of PUFA on the gut microbiota of canaries.

Finally, as for the choice of the kind of specimen, feces represent a non-invasive sampling method that can be repeated with no consequences for the host. Furthermore, unnecessary handling of the animals was avoided. Although maybe not fully representative of all the ecological niches of the intestine’s bacterial communities (i.e., duodenum, jejunum, ileum, cecum, colon, and cloaca), feces can be used to approach the microbial components of the gut, especially when instantly frozen at least at −80 °C [84,85]. It is noteworthy to point out that feces in birds go through the cloaca, which is a compartment gathering the bacterial components from gastrointestinal, reproductive, and urinary systems, and is therefore considered relevant to the health of all the systems involved [40,86].

In conclusion, our study provides a useful reference for the analysis of microbiota changes in the reproductive tract of avian species. The vast presence of Lactobacillaceae is consistent with the available literature on pet birds and may provide a further impulse for new studies on their role, both in relation to gut and reproductive health. For instance, the consistent presence of *L. aviarius* during all the phases may be a starting point for the development and testing of probiotics specific for canaries. *Lactobacillus* species (including *L*. *aviarius*) were already tested both in vitro and in vivo in hens as a probiotic, demonstrating positive effects on intestinal absorption via increased epithelial proliferation and a higher number of villus wrinkles [46]. Given the increasing availability of metagenomic technologies, screening tests may also be developed. Proteomics and metabolomics may be incorporated as well in future research to fully understand the role of the main bacterial components in the lower reproductive tract of canaries, and the influence of their products on the host health. In the present study, the time factor was considered for the first time in canaries to assess variability in the bacterial communities’ composition, showing that the gut bacterial microbiota is responsive to breeding phases in females. Interestingly, such shift was observed so far only in male passerines, and not in females, suggesting a species-specific peculiarity that should be further investigated [10]. Thus, future research will be possibly centered on the evaluation of male gut bacterial microbiota, aiming to assess the presence of microbial fluctuations along one male reproductive cycle and to make a comparison with females. It would also be interesting to repeat the sampling along with a hormonal evaluation of the individuals, in order to define the role of the physiological changes involving the host in relation to the bacterial communities’ composition. This paper lays the groundwork for a clearer understanding of canaries’ ecology and physiology. The ecology of the canaries, intended as the interaction between host and bacterial microbiota, may help improve the female canaries’ health, with important consequences for multiple fields including reproductive science, conservation, and commercial breeding of canaries.

## Figures and Tables

**Table 1:** The number of samples taken for each phase of the reproductive cycle is shown.

Groups	A	B	C
*Reproductive phase*	Parental care	Molting	Rest
*N. of samples*	12	12	11

**Table 2:** Alpha diversity indexes comparisons between groups. The *p*-values for each comparison (A vs. B, A vs. C, and B vs. C) are reported for Pielou’s Evenness, Faith phylogenetic diversity, Observed Features, and Shannon diversity indexes. The *p*-values were calculated via the PERMANOVA test. Values of *p* < 0.05 are shown in bold.

	A vs. B	A vs. C	B vs. C
*Pielou’s Evenness*	0.622461	**0.048900**	0.122800
*Faith phylogenetic diversity*	**0.026716**	0.218355	**0.009493**
*Observed Features*	**0.022741**	**0.056219**	**0.009453**
*Shannon*	0.423656	**0.042254**	**0.045201**

**Table 3:** Beta diversity indexes comparisons between groups. The *p*-value for each comparison (A vs. B, A vs. C, and B vs. C) is reported for Bray-Curtis dissimilarity and Unweighted Unifrac diversity indexes. The *p*-values were calculated via the PERMANOVA test. Values of *p* < 0.05 are shown in bold.

	A vs. B	A vs. C	B vs. C
*Bray-Curtis dissimilarity*	0.863137	**0.002997**	**0.011988**
*Unweighted Unifrac*	**0.001998**	**0.005994**	**0.003996**

**Table 4:** Gut bacterial microbiota in pet birds. The main components of the gut bacterial microbiota at the Family or Genus level in adult passerine and psittacine species kept as pet birds are reported. Feces were used as the sample in all the papers cited in the table.

	Species	Prevalent Microbiota Components	Reference
**Passerines**	**Canary** (*Serinus canaria*)	Lactobacillus, Clostridium	[19]
		Lactobacillaceae	[18]
	**Bengalese finch** (*Lonchura striata domestica)*	Lactobacillaceae, Campylobacteraceae	[10]
		Lactobacillaceae	[56]
	**Zebra finch** (*Taeniopygia guttata*)	Lactobacillaceae Campylobacteraceae	[10]
		Campylobacteraceae	[56]
		Lactobacillaceae, Campylobacteriaceae, Bifidobacteriaceae	[57]
**Psittacines**	**Cockatiel** (*Nymphicus hollandicus*)	Erysipelotrichaceae, Lachnospiraceae, Clostridiaceae	[18]
		Erysipelotrichaceae, Lachnospiraceae, Mycoplasmataceae	[57]
		Erysipelotrichaceae, Lactobacillaceae	[58]
	**Budgerigar** (*Melopsittacus undulatus*)	Lactobacillaceae	[18]
	**Lovebird** (*Agapornis* spp.)	Lactobacillaceae	[57]
	**Rose-ringed Parakeet** (*Psittacula krameri*)	Lactobacillaceae, Leuconostocaceae	[57]
	**Red-rumped parrot** (*Psephotus haematonotus*)	Lactobacillaceae	[57]

## Data Availability

Data are available from the authors upon reasonable request.
